# Pathway Network Analysis of Complex Diseases Based on Multiple Biological Networks

**DOI:** 10.1155/2018/5670210

**Published:** 2018-07-30

**Authors:** Fang Zheng, Le Wei, Liang Zhao, FuChuan Ni

**Affiliations:** College of Informatics, Huazhong Agricultural University, Wuhan 430079, China

## Abstract

Biological pathways play important roles in the development of complex diseases, such as cancers, which are multifactorial complex diseases that are usually caused by multiple disorders gene mutations or pathway. It has become one of the most important issues to analyze pathways combining multiple types of high-throughput data, such as genomics and proteomics, to understand the mechanisms of complex diseases. In this paper, we propose a method for constructing the pathway network of gene phenotype and find out disease pathogenesis pathways through the analysis of the constructed network. The specific process of constructing the network includes, firstly, similarity calculation between genes expressing data combined with phenotypic mutual information and GO ontology information, secondly, calculating the correlation between pathways based on the similarity between differential genes and constructing the pathway network, and, finally, mining critical pathways to identify diseases. Experimental results on Breast Cancer Dataset using this method show that our method is better. In addition, testing on an alternative dataset proved that the key pathways we found were more accurate and reliable as biological markers of disease. These results show that our proposed method is effective.

## 1. Introduction

The growth in knowledge of large-scale transcriptomic and proteomic technologies has enabled the identification of risk factors of complex diseases, personalized medicine, and so forth. Many algorithms (such as the supervision, nonsupervision, and statistics method) have been developed to process these data for acquiring important biological biomarkers. However, these methods still have some limitations and challenges. First, transcriptomic data analysis is the inherent complexity of multiple biological processes. Second, the data from different platforms also lead to noise. Although some methods were used to reduce the deviation, it is difficult to obtain robust result of these data. To circumvent these limitations, some computational methods project gene expression data into a molecular signaling network, but errors generated by variations in experiment also affect the accuracy to distinct different samples.

Biological networks are powerful resources for the discovery of genes and genetic modules that drive disease. Fundamental to network analysis is the concept that genes underlying the same phenotype tend to interact; this principle can be used to combine and to amplify signals from individual genes [1], a biological pathway which plays an important role in understanding the mechanisms of complex diseases, improving clinical treatment, and discovering drug targets and biomarkers [2]. The increasing availability of high-throughput biological data of complex diseases and the development of various biological networks provided better conditions to build accurate pathway analysis models. But due to a lack of abundant pathway knowledge, most pathway analysis results are incomplete, unreliable, or inaccurate [3]. So, it is an important work to build pathway analysis method of multitype data, such as gene expression data, transcriptomic data, and protein data. The major advantage of pathway-based methods is their capability to perform biologically relevant dimension reduction as a result of the analysis [4].

There are some popular pathway analysis tools, such as GSEA, SPIA, DAVID, and Pathologist. They provide different methods to explain the function of the pathway analysis.

GSEA (Gene Set Enrichment Analysis) [5] processes expression profile data with labels, sorting every pathway according to enrichment statistics of the difference of gene.

SPIA (Significant Pathway Inference Analysis) [6] combines the difference expression and pathway structure information. The effect of alteration of gene expression at different positions in the pathway is considered to be different.

DAVID [7] (Database for Annotation, Visualization and Integrated Discovery) can provide a comprehensive functional annotation of the gene list to help researchers understand the biological significance of genes behind. At present, it is the most widely used method of gene function annotation.

Although many of the above methods have shown encouraging results for finding new information, there are still some limitations. In those methods, pathway is simplified into a simple set of genes, treating pathways as unstructured sets of genes, ignoring the functional relationship between different genes in the pathway, so it cannot accurately assess function change of the related pathways.

To overcome the above limitations, new analytical methodologies are required that infer complex transcriptomic changes more accurately into the biologically network.

System biology considers that biological functions are not the result of a single gene or protein, but the interaction result of multiple biological molecules with each other. With the development of system biology, the biological network has become a powerful tool to research the complex biological activity. Biological networks can simultaneously study the interaction relationships between different biological molecules. System biology can help us understand the exercise process of biological function and explore the underlying mechanisms of biological processes.

Because changes in biological function are the result of molecular interactions, the functional annotation of differentially expressed genes should consider not only the effects of differentially expressed genes on the pathway, but also the effects of gene interaction on different pathways. In addition, consideration should also be given to the association between pathways.

So, in this paper, we proposed a network-based pathway analysis method. We find the disease related pathways through the analysis of the constructed network. The specific process for the construction of networks includes the following. First, we integrated protein-protein interaction (PPI) information and gene expression profile data into the pathway, and then the candidate genes associated with disease phenotypes were screened using mutual information calculations. Secondly, we integrated gene's GO information into pathway to calculate the correlation between the pathways and then construct the pathway network. Finally, the critical pathways are identified in the network. The experimental results of this method with breast cancer data show that our method can not only find the high risk of gene and signaling pathways, but also find an association between the risk pathways.

## 2. Methods

**Figure 1:**
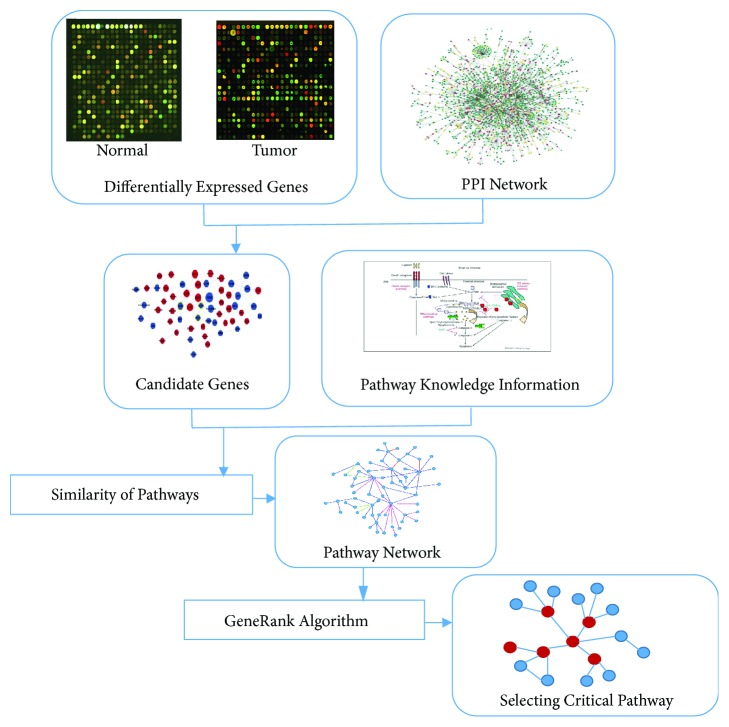
Flow chart of analysis method.

Figure 1 describes our network-based pathway analysis method. First, the differentially expressed genes were identified by comparing the disease samples and normal samples, and then they were projected onto protein interaction data. If two protein nodes all appear in differentially expressed genes, they are preserved, and the candidate genes were screened out. Secondly, the biological signaling pathways are obtained from database MSigDB which includes 1329 sets of biological metabolism and signaling pathways. The candidate genes are projected into the pathway, and we calculate the active score of each pathway for every sample according to the document [8]. Then according to the activity vectors of each biological pathway, combining the phenotypic information of the samples, the mutual information between the activity vectors and the phenotypic vectors of the samples is calculated. Next, the semantic similarity of differentially expressed genes in each pathway is calculated using gene's GO knowledge, and we calculate the correlation between the pathways and then construct the pathway network. At last, we used GeneRank algorithm to find the critical pathways in the network. In the following, the detailed explanations of our proposed method are described.

### 2.1. Project the Candidate Gene to the Pathway

Gene expression data often aim to identify genes that are differentially regulated across different classes of samples, for example, finding the genes affected by a treatment, or finding marker genes that discriminate diseased from healthy subjects. Using gene expression profiles obtained from a number of genes for several samples or experimental conditions, we can obtain a gene set that shows a differential expression pattern across different samples. However, a differential expression gene set does not guarantee the existence of a real interaction between the corresponding proteins. Instead, it only suggests that there may be an interaction between the proteins. To accurately describe the change in gene interactions for several samples or experimental conditions, here we screen genes with PPI network.

**Figure 2:**
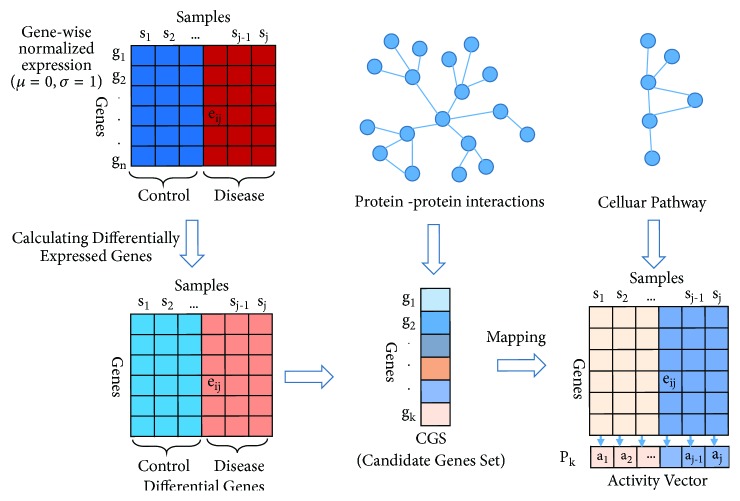
Project the candidate gene to the pathway.

In the PPI network, if the two nodes (gene) of the edge are both in the set of differentially expressed genes, then the two genes were reserved; otherwise they were removed. So we get the candidate gene set (CG set). The biological pathway is a complete metabolic pathway which contains all genes that constitute a set. The gene-gene interaction of pathway is different in different tissues or samples. These differences may be caused by genes interactions of the pathway or neighbor pathway. To find the related biological pathway of the disease, we analyze the expression of each gene set in different sample, calculating the activity score of the biological pathway (active fraction) according to [8]; main procedures are as follows and shown in Figure 2.

(1) We get the PPI data from HPRD (human protein reference database) and gene expression data of the breast cancer from NCBI GEO (the Gene Expression Omnibus at the National Center for Biotechnology Information (NCBI)).

(2) We compute differential expression genes in samples and filter through PPI network to get the candidate gene set (CGS).

(3) The biological signaling pathways are obtained from database MSigDB c2 (canonical pathway) which includes 1329 sets of biological metabolism and signaling pathways. The candidate genes are projected into the pathway, calculating the active score of each pathway.

(4) In the GEO data, we divide the samples into disease and control parts and normalize gene expression profiles of the samples and then calculate to get the differentially expressed genes. We map differential gene to the PPI network to obtain the candidate gene set (CGS). The activity scores are calculated according to the expression value of candidate gene set (CGS) on each sample. Gene expression value of gene in biological pathway for every sample constitutes an expression value matrix; each column represents a sample and each row represents a gene. A biological pathway includes* p *genes whose expression values can form a matrix of* p *rows. Each biological path corresponds to an activity vector, and the dimension of the vector is the number of samples; that is, in each sample* j*, an activity score can be calculated. The calculation formula is as follows:

(1) $$\begin{array}{c} a_{tj}=\overset{p}{\underset{i=1}{\sum }}\frac{e_{ij}}{\sqrt{p}} \end{array}$$

*a*_*tj*_ represents the activity score of the* t* biological pathway in the* j* sample, and *e*_*ij*_ represents the gene expression value of the* i* gene in the* t* biological pathway of the sample* j*, and* p* is the number of genes in the biological pathway. After that, we get an activity vector of biological pathways; vector  [*a*_*t*1_, *a*_*t*2_, *a*_*t*3_,…*a*_*tm*_] represents the activity score of the* t *biological pathway in* m* samples.

Next, a phenotypic vector is constructed based on the phenotypic labels of the samples, and then the mutual information between the activity vectors and the phenotypic vectors of the samples is computed by combining the activity vectors of each biological pathway.

### 2.2. Mutual Information With Phenotype

**Figure 3:**
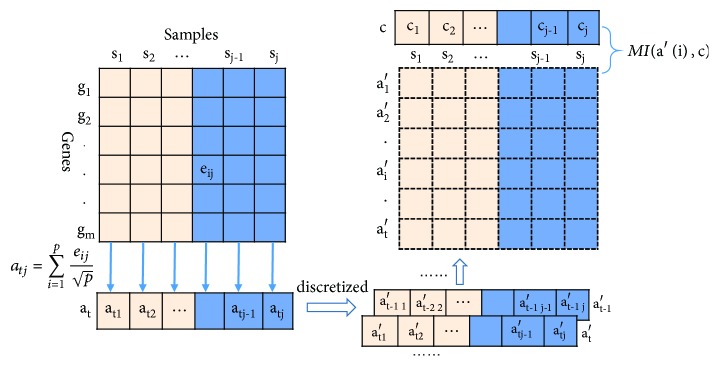
Calculation of mutual information.

Mutual information (MI) is a commonly used method of information measurement in information theory. In [8], the rationale behind using MI to classify cancer patients is explained, and the processing is shown in Figure 3. By calculating the mutual information between the activity vectors and the phenotypic vectors of biological pathways, the correlations between the two vectors are measured, that is, the influence of a biological pathway on the phenotype of the disease.

Constructing a phenotype vector based on the phenotype of the sample, [*c*1, *c*2, *c*3,…*cm*], the phenotypic vector is a zero-one vector, and if the sample is tumor, the corresponding value is one, otherwise zero.

Using *a*(*i*) to indicate the activity score of the* i *biological pathway on each sample, *a*(*i*)=[*a*_*i*1_, *a*_*i*2_, *a*_*i*3_,…*a*_*im*_]. *c* is used to represent the phenotypic vectors of* m *samples, *c* = [*c*_1_, *c*_2_, *c*_3_,…*c*_*m*_]. So the correlation between biological pathways and disease phenotype *S*(*i*) can be represented by mutual information *MI*(*a*′(*i*), *c*) between *a*′(*i*) vector and *c*  vector. *a*′ is* a* discretized form of* a*. The formula is as follows:

(2) Mi=MIa′i,c=∑x∈a′∑y∈cpx,ylog⁡px,ypxpy

The activity score *a* is discretized into ⌊log⁡2(*sum*  *of*  *smaples*) + 1⌋ equally spaced bins to obtain *a*′, respectively, *p*(*x*, *y*) is the joint probability density function of *a*′  and *c*, and *p*(*x*)  and  *p*(*y*)  are the marginal probability density function of *a*′ and *c*.

## 3. Constructing Pathway Network Related To Disease

The interaction of the pathway can be represented as a network which was constructed as follows.

Let *G*(*V*, *E*) comprise a set *V* of pathways and a set *E* denote the weighted pathway-pathway interaction network with *E* ∈ *V∗V*. Here, we use similarity to define weight of the network. *V* = {*p*_1_, *p*_2_ … *p*_*n*_}, *n* = |*V*| is the number of pathways. Matrix *A* represents the weighted *n* × *n* adjacency matrix of *G*, where *w*_*ij*_ denotes the weight of the edge connecting pathway *p*_*i*_ to *p*_*j*_ and *w*_*ij*_ is calculated by the similarity of *p*_*i*_ and *p*_*j*_. Supposing two pathways *p*_1_and *p*_2_, *p*_1_, *p*_2_ is a set of genes. *p*_1_ = {*g*_11_, *g*_12_ … *g*_1*n*_}, and  *p*_2_ = {*g*_21_, *g*_22_ … *g*_2*m*_}.The similarity of *p*_1_ and *p*_2_ can be calculated as weight of network. The similarity between the two pathways is the ratio of the sum of the similarities of all similar genes to the sum of the two pathway's elements. The formula is shown in

(3) $$\begin{array}{c} {sim}_{pi,pj}=\frac{\sum_{g_{ix\in pi},g_{jy\in pj}}sim\left(g_{ix,}g_{jy}\right)}{M+N} \end{array}$$

*M* = |*p*_*i*_| is the number of genes on *p*_*i*_, and *N* = |*p*_*j*_| is the number of genes on *p*_*j*_.

By this formula, the similarity values of each pair of candidate pathways can be calculated, and the pathway network can be constructed to identify the critical pathway.

We use GOSemSim package of R language [9] to calculate the semantic similarity (*sim*(*g*_*ix*_, *g*_*jy*_)) between genes, including molecular function similarity (MF), biological processes similarity (BP), and Cellular Component (CC) similarity. Since a pathway contains multiple genes, we calculate the similarity of genes on the pathway as the similarity between the pathways to obtain the pathway similarity matrix *sim*_*pi*,*pj*_.

## 4. Identifying Significant Pathways of Pathway Network

Based on the biological pathway information and the similarity information of genes in the pathway, we constructed the biological pathway interaction network. In the network, each node represents a biological pathway, and if the two pathways contain differentially expressed genes, then they are connected. And the similarity of the differential genes on the two pathways is used as the similarity of the two pathways.

After constructing the biological pathway network, we hope to find biological pathways related to cancer phenotype. We use the random walk algorithm combined with phenotypic information to find the key nodes in the pathway network. The GeneRank method is a sorting algorithm proposed by Morrison et al. [10]. In our method, the initial node order is determined by the value of mutual information* MI*, and the transfer matrix is obtained by the pathway similarity matrix (*sim*_*pi*,*pj*_). According to the random walk GeneRank algorithm, starting from the initial node, the final stable pathway node sequence is obtained by iterative calculation of the transfer matrix. This approach considers both the pathway and phenotype information and the semantic similarity between pathways, thus avoiding the node as isolated individuals and ignoring the important nodes that are highly correlated with other nodes. Therefore, this method can find potential pathway nodes.

We let set *P* = {*p*_1,_*p*_2,_ …, *p*_*N*_} represent *n* nodes in the pathway network. According to whether there is a similarity relationship between the two pathway nodes, the adjacency matrix *w*(*i*, *j*) can be obtained by a similar matrix *sim*_*pi*,*pj*_ according to a threshold *θ*, as shown in

(4) $$\begin{array}{c} w\left(\;i,j\;\right)=\left\{\begin{array}{cc} 1 & if\;\;sim\left(pi,pj\right)\geq \theta \\ 0 & others \end{array}\right. \end{array}$$

Sort the pathway set *P* according to the mutual information (*MI*) of pathway and phenotype. The initial row rank of *P* is obtained, which is denoted as *x*, *ex* = (*ex*_1,_*ex*_2,_ …, *ex*_*N*_).

According to the definition,* W* is a symmetric matrix, *w*_*i*,*j*_ = *W*(*i*, *j*) = *W*(*j*, *i*) = *w*_*j*,*i*_. According to graph theory, the degree of the i node is equal to the sum of the elements of row i of matrix *w* and is expressed in  deg_*i*_, as shown in

(5) $$\begin{array}{c} {deg}_{i}=\overset{n}{\underset{j=1}{\sum }}w_{i,j}=\overset{n}{\underset{j=1}{\sum }}w_{j,i} \end{array}$$

*d* is the damping coefficient, and the closer the value of* d* is to 0, the greater the impact of the mutual information is on the node sorting; on the contrary, if* d* is closer to 1, node sorting is more affected by the similarity. In our algorithm, we initialized* d* to 0.85.

The algorithm steps are as follows:

*Input*: initialization vector *ex* = (*ex*_1,_*ex*_2,_ …, *ex*_*N*_); adjacency matrix *w*; parameter* d.*

*ε* is the error value; max is the maximum iterations.

*Output: r*;

(1) Data Preprocessing:
  (6) r0=exex1;rn=r1n,r2n,…,rNn;n=0,1,2,….
(2) Iteration:
  (7) rjn=1−dexj+d∑i=1Nwijrin−1degi,1≤j≤N;resn=rn−rn−11;
(3) Stop Condition
If *res*^[*n*]^ ≤ *ε*  *or*  *n* ≥ *max*  , *Stop*;
Else goto (2);
Return *r*=*r*^[*n*]^;

## 5. Experiments and Results

### 5.1. Data

The Breast Cancer Dataset was downloaded from GEO (Gene Expression Omnibus) website (https://www.ncbi.nlm.nih.gov/geo/), including GSE33447 [11], GSE9309, GSE15852[12], GSE5364 [13]. and GSE20437 [14]. The dataset consists of 484 samples obtained from comparing 387 breast cancer samples with 97 normal samples, as shown in Table 1.

The gene expression profiles of 369 cases of breast cancer and 73 cases of normal breast tissues were obtained, and the differentially expressed genes were analyzed, PPI network was obtained from the Human Protein Reference Database (http://www.hprd.org/) [15]. The pathways were downloaded from the Molecular Signatures Database website (http://software.broadinstitute.org/gsea/msigdb) [16]. The database mainly collects gene set which was annotated with certain biological functions. We chose C2 gene sets (canonical pathways, 1392 gene sets); the set of genes is derived from several major biological pathway databases, including BioCarta [17], KEGG [18], and Reactome [19] databases.

To identify the significance of the given pathway, first, we computed differential expression genes of the sample datasets (GSE9309, GSE15852) and then dealt with the PPI data. PPI network was mapped into the differential expression gene; we obtained candidate gene set (CGS). Secondly, the candidate genes are mapped into the pathways, calculating the active score of each pathway, and then the mutual information is computed by combining the activity vectors of each biological pathway. Finally, we calculate the similarity of the pathway to obtain pathway network, and the random walk algorithm combined with phenotypic information was used to find significance node of the pathway network.

To provide a more comprehensive understanding of the proposed method, we discuss the method from the following aspects separately.

### 5.2. The Results of Pathway Recognition

**Figure 4:**
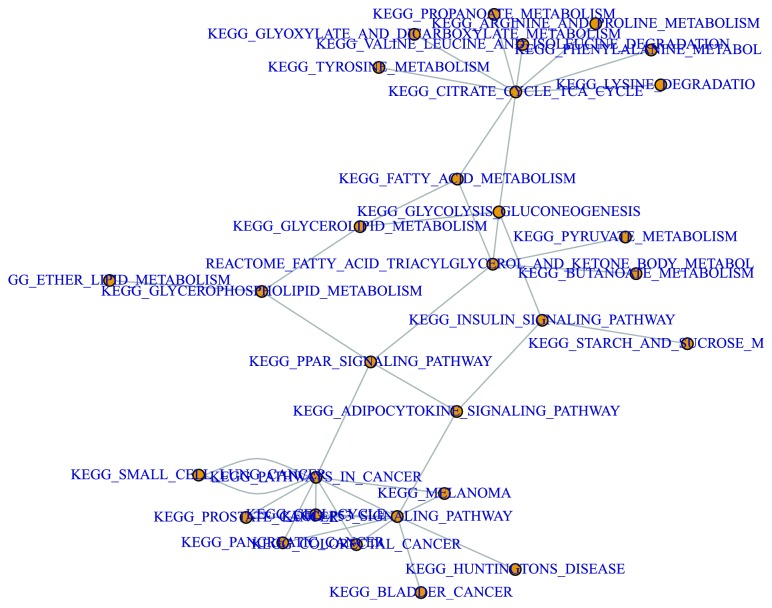
Interaction between disease pathways.

According to the above description, the rank of each pathway is the degree of relevance between the given pathway and the corresponding disease. The rank is calculated by algorithm (see in Section 4). In this study, the significance of the pathway was tested by hypergeometric distribution of each pathway with annotated differential genes using formula (8). The top 15 pathways with p-value are shown in Table 2; we selected part of the interaction pathways, as shown in Figure 4.

(8) $$\begin{array}{c} p-value=1-\overset{x-1}{\underset{i=0}{\sum }}\frac{\left(\begin{array}{c} M\\ i \end{array}\right)\left(\begin{array}{c} U-M\\ N-i \end{array}\right)}{\left(\begin{array}{c} U\\ N \end{array}\right)} \end{array}$$

 
*U*—the number of genes in the human genome;
 
*N*—the number of differential genes;
 
*M*—the number of genes in the pathway;
 
*x*—the number of differentially expressed genes in this pathway.

The top 1 pathway is KEGG_FATTY_ACID_METABOLISM; the pathway is supported by [20–22], and the KEGG_SPLICEOSOME [23–25], REACTOME_ METABOLISM_OF_NON_CODING_RNA [26, 27], and REACTOME_CELL_CYCLE [28] are all very important metabolic pathways, and we focus on the following important pathways.

One significant pathway identified by our method was P53_SIGNALING_PATH-WAY [29–31]. Currently, breast cancer is the most prevalent cancer diagnosed in women, with an estimated 1.8 million cases reported worldwide in 2013 [32, 33]. Radiation is commonly adopted as an adjuvant therapy for the management of breast cancer [34]. However, there is growing evidence that autophagy is induced by ionizing radiation, and this induction plays a crucial role in radiosensitivity [35, 36]. Furthermore, the regulatory effect of autophagy in radiation-induced cell death remains controversial, and the underlying molecular mechanisms remain to be fully characterized. The research of [37] shows that the p53/DRAM signaling pathway appears to contribute to radiation-induced autophagic cell death in MCF-7 breast cancer cells.

Another significant pathway was KEGG_PPAR_SIGNALING_PATHWAY. In [38], the authors analyzed six pathological complete response (pCR) patients and 25 patients with non-pCR; 300 probes (231 genes) were identified as differentially expressed between pCR and residual disease by the SAM program when the fold change was >2. The gene functional enrichment analysis revealed 15 prominent gene categories that were different between pCR and non-pCR patients, most notably the genes involved in the peroxisome proliferator-activated receptor (PPAR), DNA repair, and ER signal pathways and in the immune-related gene cluster; they believe that the PPAR pathway may be an important predictor of genes that are involved in the chemotherapy response.

The other pathway was PID_SHP2_PATHWAY; in [39], the researchers show a fundamental role for Src-homology 2 domain-containing phosphatase 2 (SHP2) in these processes in human epidermal growth factor receptor 2- (HER2-) positive and triple-negative breast cancers. Knockdown of SHP2 eradicated breast tumor-initiating cells in xenograft models, and SHP2 depletion also prevented invasion in three-dimensional cultures and in a transductal invasion assay in vivo. Notably, SHP2 knockdown in established breast tumors blocked their growth and reduced metastasis. Mechanistically, SHP2 activated stemness-associated transcription factors, including v-myc myelocytomatosis viral oncogene homolog (c-Myc) and zinc finger E-box binding homeobox 1 (ZEB1), which resulted in the repression of let-7 microRNA and the expression of a set of “SHP2 signature” genes. They found these genes to be simultaneously activated in a large subset of human primary breast tumors that are associated with invasive behavior and poor prognosis. These results provide new insights into the signaling cascades influencing tumor-initiating cells as well as a rationale for targeting SHP2 in breast cancer.

**Figure 5:**
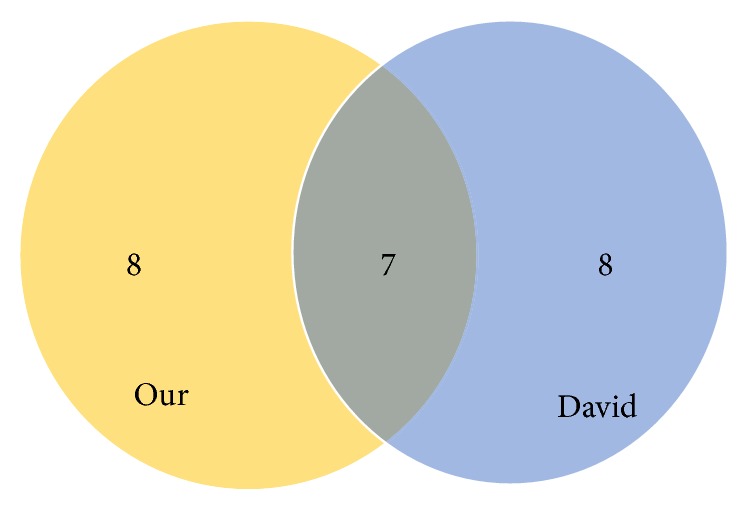
DAVID method and our method to identify the risk pathway.

Moreover, we obtained top 15 pathways; their p-values are all less than 0.05. In order to test the effectiveness of this method, we compare with the most used DAVID software. The identified top 15 pathways are compared with DAVID method and our method to identify the risk pathway of breast cancer as shown in Figure 5; the common pathways are shown in Table 3.

DAVID is a widely used and approved method. According to the pathway set we have identified (in Tables 2 and 5). Pathways identified by our method and DAVID method have obvious intersection (in Figure 5); the p-value of the pathway is also significant. In addition, compared with DAVID, we also found some pathways which DAVID did not find. In Table 2, PID_BARD1_PATHWAY [40–45], PID_SHP2_PATHWAY [39], REACTOME_TRANSPORT_OF_MATURE_TRANSCRIPT_TO_CYTOPLASM [46], and REACTOME_METABOLISM_OF_NON_CODING_RNA [46, 47] are the pathways that our method identified but DAVID did not. The relationship between them and breast cancer has been evaluated in detail and is affirmative in the corresponding literature.

So, according to the above analysis, it can be concluded that our proposed method is effective in identifying the important pathways of the complex diseases.

### 5.3. Test on Dataset

#### 5.3.1. Results and Analysis

**Figure 6:**
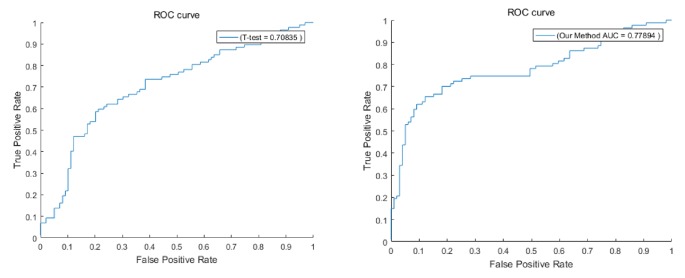
Test on dataset.

To estimate the classification performance, firstly we prepared our dataset (GSE9309, GSE15852) and took 80 genes in the selected pathways as features, and SVM [48] is employed to classify the selected samples. Next, a 10-fold cross-validation was used to train and test SVM. The above experiment was repeated 100 times; the average value of the 100-time calculation is taken as the final result. In order to evaluate our method, we compared the classification results of cancer and normal samples with the commonly used methods based on differential expression. We choose the T-test method, and the T-test can be used to test whether the means of two independent normal distribution samples are equal. For gene expression data, we can test whether there is a significant difference in the expression of a gene between different phenotypes, that is, to identify differentially expressed genes through tests. The results obtained by the T-test method are compared with our method according to the genes contained in the biological pathway, and the same number of genes is sorted according to the order. The comparison results show that the method is superior to the T-test method, and the experimental results show that our proposed method is more effective in distinguishing cancer from normal samples, and the results are shown in Figure 6.

**Figure 7:**
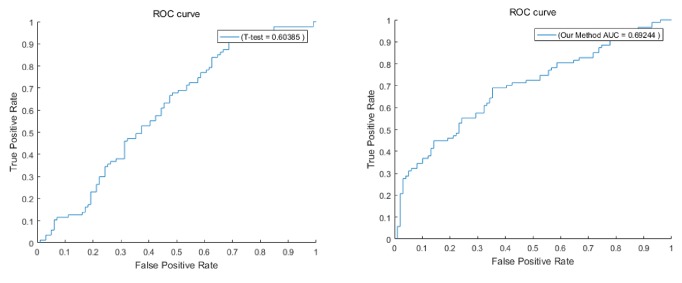
Test on independent datasets.

We applied the feature gene set to the independent gene expression datasets (GSE5364, GSE33447, and GSE20437). This set of data is not related to the data previously used, and they are independent of each other. In order to evaluate our proposed method objectively, we used the same number of biological pathways or gene markers to compare the results. The biological pathway markers obtained by our method have a good discrimination in the dataset, higher than 0.6, close to 0.7, and the results are shown in Figure 7. However, the AUC of the currently reported prognostic models on independent datasets is very difficult to reach 0.7 [49], which shows the superiority of our method.

#### 5.3.2. Test on Other Datasets

Secondly, in order to test the robustness of our method, we apply the method in this article to four gastric cancer gene expression datasets. The dataset was also downloaded from GEO, which includes GSE63089 [50], GSE56807 [51], GSE33335 [52], and GSE19826 [53]. PPI data was obtained from STRING (https://stringdb-static.org/); the sources of other data are the same. The dataset consists of 177 samples obtained from comparing 87 tumor samples with 90 normal samples, which is shown in Table 4; the top 15 pathways with p-value are shown in Table 5.

**Figure 8:**
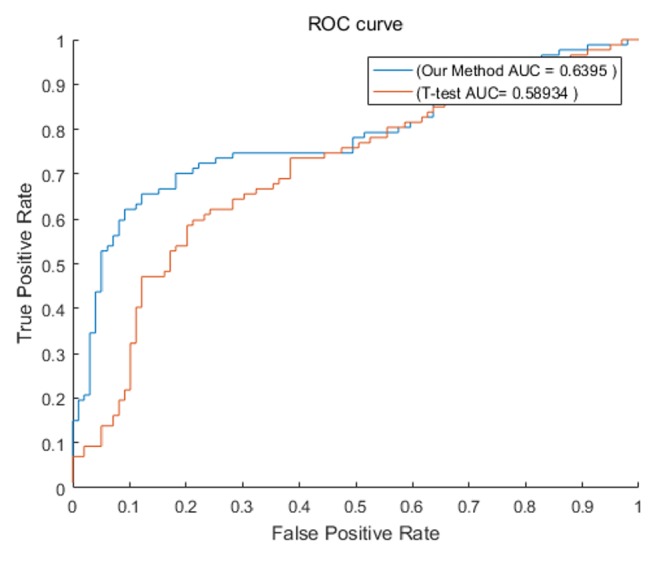
Test on independent gastric cancer dataset.

We used our method to test on the gastric cancer dataset, and we selected the same number of biological pathways and gene markers. Compared with the T-test, our method has also achieved a higher or comparable value. We used GSE63089 and GSE56807 as the training set, with GSE33335 and GSE19826 as the test set. A 10-fold cross-validation was used to train and test SVM. The above experiment was repeated 50 times; the average value is taken as the final result. Our method also has good discrimination in this dataset, higher than 0.6 and better than T-test; the results on gastric cancer data are shown in Figure 8.

### 5.4. Conclusions

Complex diseases, especially cancer, are extremely harmful to human health. Therefore, the identification of cancer markers is the key of the study. Pathway analysis combined with multiple types of high-throughput data reflects the biological processes more clearly. Therefore, pathway-based complex disease analysis method has become a hot research topic. Unlike previously available pathway analysis methods, we have considered not only the genes interaction of the pathway, but also the interaction between the pathways.

In this paper, we proposed a new approach to consider the correlation between biological pathways and establish a biological pathway interaction network. Then, the GeneRank algorithm based on random walk and the mutual information of phenotype were used to select cancer related biological pathways. Finally, we use the support vector machine and feature selection method to apply to cancer datasets. The results show that our method achieves better results than T-test method. In addition, the validation in the independent dataset and the functional analysis of the biological pathway indicate that the pathway we identified as a biological marker of disease is more accurate and reliable. We will employ more datasets to assess the validity of our approach in future research.

## Figures and Tables

**Table 1:** Breast Cancer Data Set.

DataSet	Normal	Tumor
GSE 9309	9	132
GSE 15852	43	43
GSE 5364	13	186
GSE 33447	8	8
GSE 20437	24	18

**Table 2:** Top 15 pathways identified with our method.

Rank	Pathway Name	gene	p-value
1	KEGG_FATTY_ACID_METABOLISM	42	5.76e-12
2	KEGG_STARCH_AND_SUCROSE_METABOLISM	52	0.0047780
3	KEGG_SPLICEOSOME	128	3.63e-12
4	KEGG_PPAR_SIGNALING_PATHWAY	69	8.64e-21
5	KEGG_P53_SIGNALING_PATHWAY	69	0.0005045
6	KEGG_ADIPOCYTOKINE_SIGNALING_PATHWAY	68	2.23e-09
7	PID_SHP2_PATHWAY	58	2.40e-05
8	PID_BARD1_PATHWAY	29	0.0010373
9	REACTOME_MRNA_3_END_PROCESSING	36	0.0137007
10	REACTOME_METABOLISM_OF_NON_CODING_RNA	49	0.0008123
11	REACTOME_CELL_CYCLE	421	1.02e-08
12	REACTOME_SIGNALING_BY_BMP	23	0.0247461
13	REACTOME_TRANSPORT_OF_MATURE_TRANSCRIPT_TO_CYTOPLASM	54	0.0426007
14	REACTOME_CELL_CYCLE_MITOTIC	325	9.35e-07
15	REACTOME_PROCESSING_OF_CAPPED_INTRONLESS_PRE_MRNA	23	0.0127461

**Table 3:** Common pathways with our method and David.

Pathway Name	p-value
KEGG_FATTY_ACID_METABOLISM	5.76e-12
KEGG_SPLICEOSOME	3.63e-12
KEGG_PPAR_SIGNALING_PATHWAY	8.64e-21
KEGG_P53_SIGNALING_PATHWAY	0.0005045
KEGG_ADIPOCYTOKINE_SIGNALING_PATHWAY	2.23e-09
REACTOME_CELL_CYCLE	1.02e-08
REACTOME_CELL_CYCLE_MITOTIC	9.35e-07

**Table 4:** Gastric Cancer Data Set.

DataSet	Normal	Tumor
GSE 63089	45	45
GSE 56807	5	5
GSE 33335	25	25
GSE 19826	15	12

**Table 5:** Top 15 pathways identified with our method in Gastric Cancer Data Set.

Rank	Pathway Name	gene	p-value
1	KEGG_DNA_REPLICATION	36	1.50e-20
2	KEGG_PROTEASOME	48	9.07e-05
3	KEGG_ARGININE_AND_PROLINE_METABOLISM	54	0.000489
4	KEGG_GLYCEROLIPID_METABOLISM	49	5.12e-05
5	KEGG_PURINE_METABOLISM	159	1.68e-12
6	KEGG_PATHWAYS_IN_CANCER	328	2.52e-09
7	KEGG_SPLICEOSOME	128	0.0003348
8	KEGG_NUCLEOTIDE_EXCISION_REPAIR	44	0.0001406
9	KEGG_MELANOGENESIS	102	0.0481885
10	KEGG_RNA_DEGRADATION	59	0.0003626
11	KEGG_MAPK_SIGNALING_PATHWAY	267	0.0014066
12	KEGG_GLYCEROLIPID_METABOLISM	49	5.119e-05
13	KEGG_DRUG_METABOLISM_CYTOCHROME_P450	72	0.0115133
14	KEGG_CYTOKINE_CYTOKINE_RECEPTOR_INTERACTION	267	0.0183417
15	KEGG_PROSTATE_CANCER	89	0.0274642
